# Real-world experience of angiotensin receptor-neprilysin inhibitors in patients with heart failure and dialysis

**DOI:** 10.3389/fcvm.2024.1393440

**Published:** 2024-07-22

**Authors:** I-Ning Yang, Chi-Ya Huang, Chun-Ting Yang, Han-Siong Toh, Wei-Ting Chang, Li-Wei Su, Yu-Min Lin, Ming-Cheng Wang, Hsien-Yi Wang, Chia-Te Liao

**Affiliations:** ^1^ Division of Nephrology, Department of Internal Medicine, Chi Mei Medical Center, Tainan, Taiwan; ^2^ Institute of Clinical Pharmacy and Pharmaceutical Sciences, College of Medicine, National Cheng Kung University, Tainan, Taiwan; ^3^ Department of Intensive Care Medicine, Chi Mei Medical Center, Tainan, Taiwan; ^4^ Institute of Clinical Medicine, College of Medicine, National Cheng Kung University, Tainan, Taiwan; ^5^ Department of Health and Nutrition, Chia Nan University of Pharmacy and Science, Tainan, Taiwan; ^6^ Division of Cardiovascular Medicine, Chi Mei Medical Center, Tainan, Taiwan; ^7^ School of Medicine, College of Medicine, National Sun Yat-sen University, Kaohsiung, Taiwan; ^8^ Department of Internal Medicine, Chi Mei Medical Center, Tainan, Taiwan; ^9^ Division of Nephrology, Department of Internal Medicine, National Cheng Kung University Hospital, College of Medicine, National Cheng Kung University, Tainan, Taiwan; ^10^ Department of Sport Management, College of Leisure and Recreation Management, Chia Nan University of Pharmacy and Science, Tainan, Taiwan; ^11^ Division of Cardiovascular Medicine, Chi Mei Medical Center, School of Medicine, College of Medicine, National Sun Yat-sen University, Kaohsiung, Taiwan

**Keywords:** angiotensin receptor-neprilysin inhibitors, angiotensin-converting enzyme inhibitors (ACE inhibitors), angiotensin receptor blockers (ARB), heart failure and reduced ejection fraction, end-stage renal disease (ESRD), dialysis

## Abstract

**Introduction:**

Although angiotensin receptor-neprilysin inhibitor (ARNI) has shown promise in patients with heart failure and reduced ejection fraction (HFrEF), the treatment effect in HFrEF patients with end-stage renal disease (ESRD) undergoing dialysis is uncertain. This study aimed to examine the real-world effects of ARNI vs. angiotensin-converting enzyme inhibitors/angiotensin receptor blockers (ACEI/ARB) in this subpopulation.

**Methods:**

This multi-institutional, retrospective study identified 349 HFrEF patients with ESRD on dialysis, who initiated either ARNI or ACEI/ARB therapy. Efficacy outcomes included rates of hospitalization for heart failure (HHF) and mortality, as well as changes in echocardiographic parameters. Safety outcomes encompassed hypotension and hyperkalemia. Treatment effects were assessed using Cox proportional hazards models, with additional sensitivity analyses for robustness.

**Results:**

Out of 349 patients screened, 89 were included in the final analysis (42 in the ARNI group and 47 in the ACEI/ARB group). After 1 year of treatment, echocardiographic measures between the two groups were comparable. The primary composite rate of HHF or mortality was 20.6 events per 100 patient-years in the ARNI group and 26.1 in the ACEI/ARB group; the adjusted hazard ratio was 0.98 (95% CI: 0.28–3.43, *P* = 0.97). Their safety outcomes did not differ significantly. Sensitivity analyses, including repetitive sampling, propensity score matching, and extended follow-up, corroborated these findings.

**Conclusion:**

ARNI has proven effective in treating HFrEF patients; however, significant benefits were not observed in these patients with ESRD undergoing dialysis compared with ACEI/ARB in this real-world cohort. Future research employing a more extended follow-up period, larger sample size, or randomized design is warranted to investigate the treatment effects in this subpopulation.

## Introduction

Heart failure (HF) is a condition characterized by the heart's inability to effectively circulate blood throughout the body. It can be classified into three categories based on left ventricular ejection fraction (LVEF) values: heart failure with reduced ejection fraction (HFrEF), mildly reduced, and preserved ejection fraction (1, 2). HF is a clinical syndrome that may involve multiple organs, such as the liver in cardio-hepatic or the kidneys in cardio-renal syndromes (3–5). Dysfunction of these organs can exacerbate clinical symptoms and heart function, leading to increased morbidity and mortality in HF patients, and complicating treatment approaches.

Managing HF continues to pose significant challenges, with guideline-directed medical therapies primarily targeting HFrEF, as informed by clinical trial results (1, 6). Nevertheless, these trials commonly exclude HF patients with advanced chronic kidney diseases (CKD), resulting in a lack of evidence regarding the clinical efficacy of treatments for this vulnerable population (5, 7, 8). For example, the Angiotensin-Neprilysin Inhibition vs. Enalapril in Heart Failure (PARADIGM-HF) trial demonstrated the substantial benefits of angiotensin receptor-neprilysin inhibitors (ARNI) for HFrEF patients, but the effectiveness of ARNI in HFrEF patients with advanced renal dysfunction remains unclear due to insufficient evidence (9).

Previous observational studies have investigated the effectiveness of ARNI in this specific population, but the reported treatment effects have been inconsistent (10–13). For example, one study highlighted the advantages of ARNI in reducing mortality and hospitalization for HF (HHF) compared to angiotensin-converting enzyme inhibitors (ACEI) or angiotensin receptor blockers (ARB) (12). Nevertheless, another multicenter study reported minimal additional benefits for these clinical outcomes (11). Furthermore, the heterogeneity of patients in pre- and post-dialysis stages in these studies could contribute to the observational disparities in outcomes, as dialysis treatments may vary clinical presentations among patients with advanced CKD (14, 15).

Given the inconsistent clinical outcomes and the heterogeneity observed in HFrEF patients with advanced CKD, the objective of this study was to evaluate the real-world efficacy and safety of ARNI compared to ACEI/ARB in HFrEF patients concurrent with end-stage renal disease (ESRD) undergoing dialysis. The focus was on cardiovascular outcomes, all-cause mortality, adverse events and echocardiogram parameters.

## Methods

We conducted a multi-institutional retrospective cohort study to examine the association between ARNI and cardiovascular outcomes in HFrEF patients with ESRD and undergoing dialysis. This study adhered to the Declaration of Helsinki and received approval from the Research Ethics Committee of Chi Mei Hospital (IRB No.10903-E02). All patient data were de-identified during the processing stage, and the need for informed consent was waived. Our study was reported in accordance with the Strengthening the Reporting of Observational Studies in Epidemiology (STROBE) Statement guidelines for reporting observational studies.

### Patient selection

The standardized electronic health records (EHR) database from the Chi Mei Hospital system, including a medical center, a regional hospital, and a district hospital, which serves as referral medical institutions for HF management in southern Taiwan, was utilized in this study. We extracted information on patients' demographics, vital signs, laboratory data, medical history, medications, and imaging reports from the database. Diagnoses were defined using International Classification of Diseases, Ninth version, Clinical Modification (ICD-9-CM) diagnosis codes before 2016, and International Classification of Diseases, Tenth version, Clinical Modification (ICD-10-CM) diagnosis codes thereafter (Supplementary File S1).

**Figure 1:**
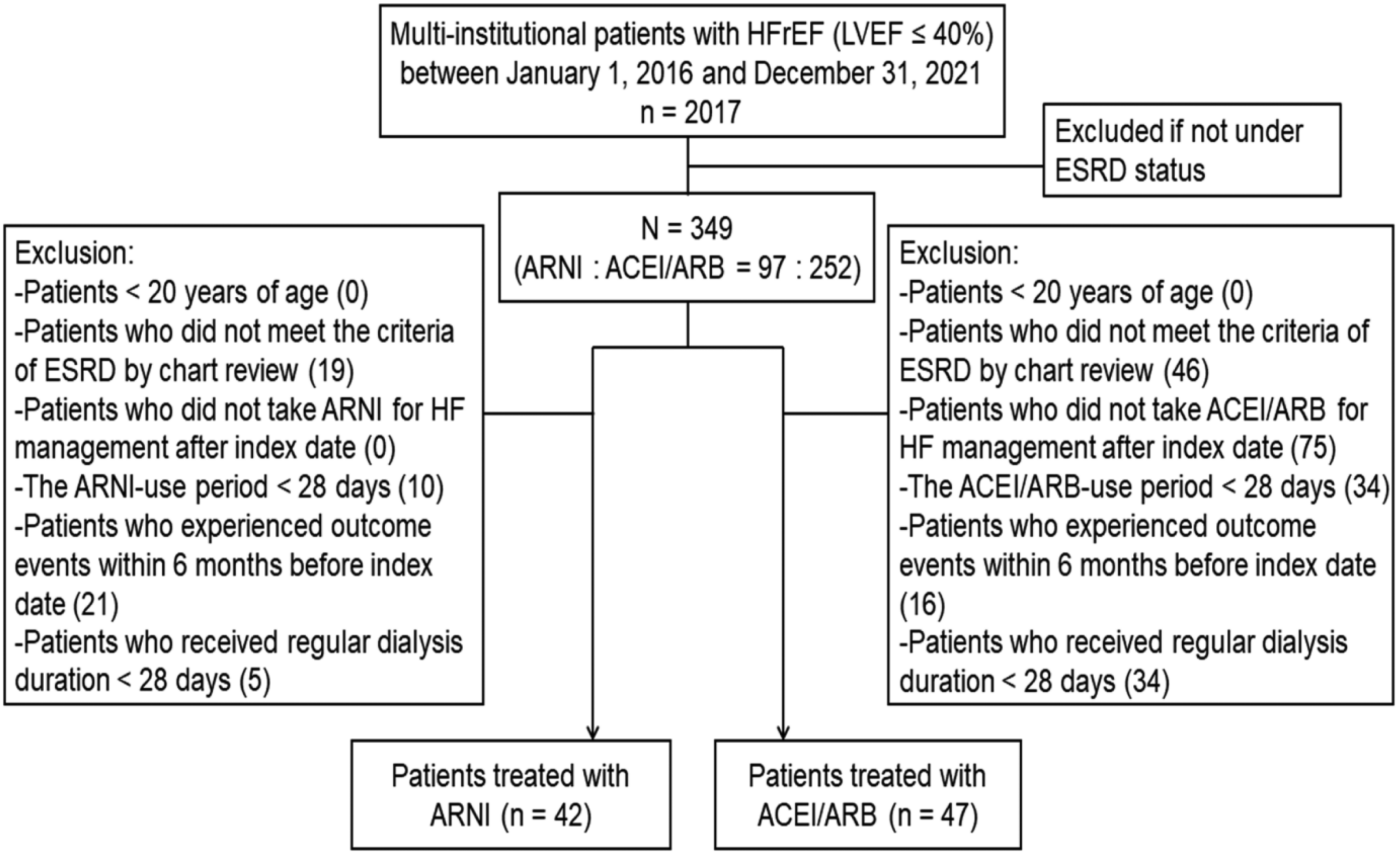
Flow chart of patient selection.

We identified HFrEF and ESRD patients from the EHR database between January 2016 and December 2021, as ARNI has been available in Taiwan since 2016. HFrEF was defined as a patient with an HF diagnosis and an LVEF below 40% (16); ESRD was defined as a patient undergoing maintenance dialysis for over 28 days (17). We defined the first ARNI prescription date as the index date for the ARNI group, and the first documented LVEF below 40% and concurrent with prescription of ACEI or ARB as the index date for the ACEI/ARB group. The baseline period was set as 1 year before the index date. We included patients aged 20 years or older, diagnosed with HFrEF and ESRD, and receiving ARNI, ACEI, or ARB within 28 days after the index date. Patients younger than 20 years and those not using ARNI, ACEI, or ARB for HF management were excluded. Additional exclusion criteria comprised those who experienced outcome events within 6 months before the index date, whose duration of ARNI, ACEI, or ARB use was less than 28 days, or who did not receive dialysis for more than 28 days. After applying the selection criteria, 89 patients were included in our final analysis (Figure 1).

### Variables

Baseline characteristics included age, sex, body mass index, vital signs, dialysis duration, laboratory data, comorbidities, prior history of HF treatments, and echocardiographic reports. Previous HF treatments encompassed guideline-directed medical therapy and interventions such as implantable cardioverter-defibrillator and cardiac resynchronization therapy. Comorbidities were determined using diagnostic codes in the inpatient databases at least once during the baseline period (Supplementary File S1).

### Follow-up and outcomes

The primary outcome was a composite of hospitalization for HF (HHF) or all-cause mortality. Secondary outcomes, in hierarchical order, included HHF, all-cause mortality, and 1-year echocardiogram data of cardiac remodeling. Safety outcomes were the episodes of post-treatment hypotension (systolic blood pressure <90 mmHg) and hyperkalemia (serum potassium ≥5 mmol/L). The follow-up period extended from the index date to the occurrence of cardiovascular events, death, 1-year follow-up, or the end of the study period (December 31, 2021), whichever came first. We also evaluated changes in heart function by echocardiogram, including LVEF, left ventricular internal diameter of end-diastole and end-systole (LVIDd and LVIDs), and left atrial diameter (LAD) from baseline and follow-up visits after the index date in both groups.

### Statistics and sensitivity analyses

Descriptive statistics were expressed as means and standard deviations (SD) or medians and interquartile ranges for continuous variables and numbers and percentage for categorical variables. We compared differences in patient baseline characteristics between the ARNI and ACEI/ARB groups using the Student's *t*-test for continuous variables and the Chi-square test for categorical variables. The Mann-Whitney *U*-test was used for continuous variables with non-normal distribution. Missing data in body mass index and laboratory results were addressed using multiple imputations by chained equations.

**Figure 2:**
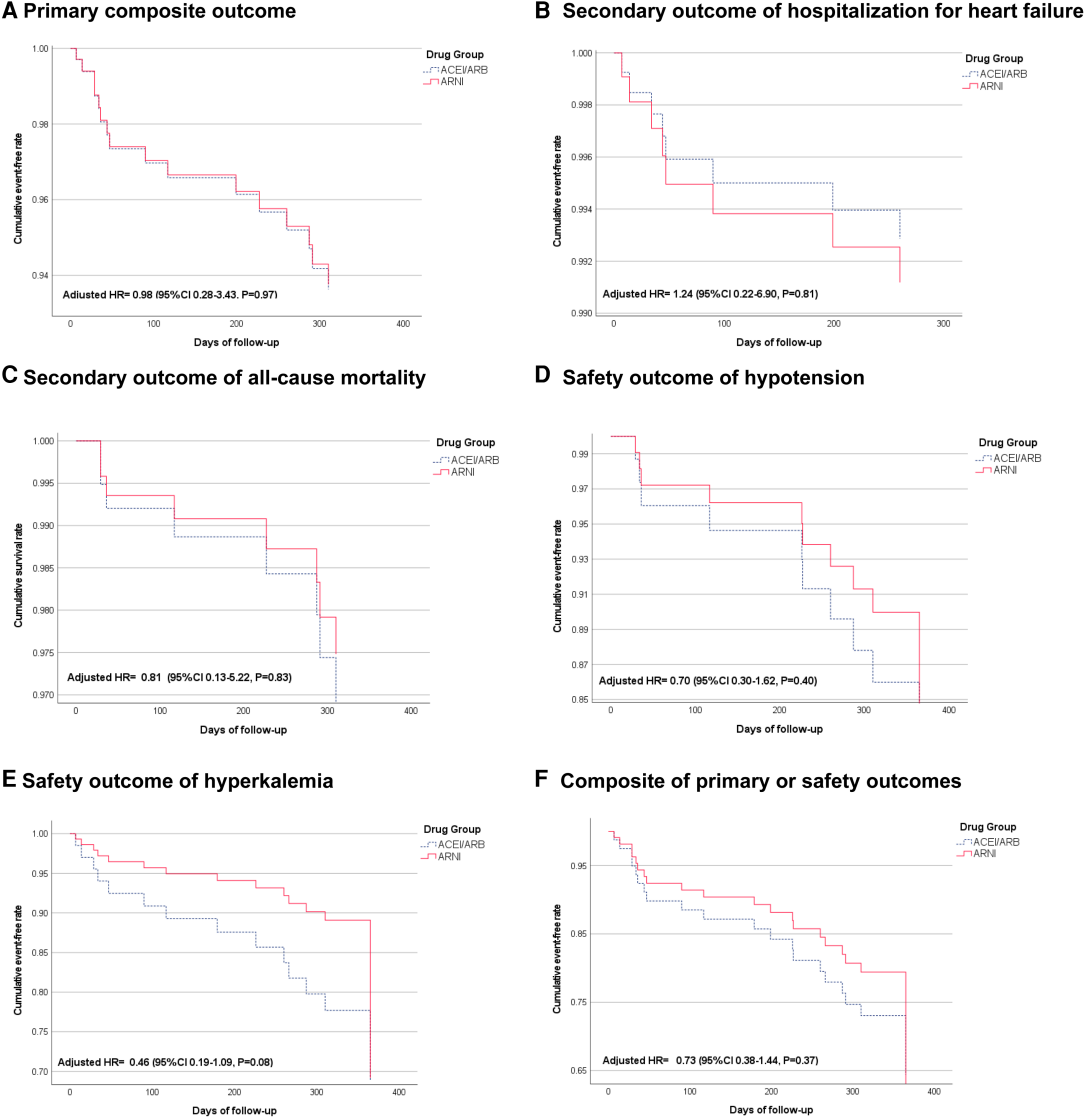
Cox-adjusted cumulative event-free rate of primary, secondary, and safety outcomes. (**A**) Shows the primary composite outcome of hospitalization for heart failure or all-cause mortality. (**B**) Shows the secondary outcome of hospitalization for heart failure. (**C**) Shows the secondary outcome of all-cause mortality. (**D**) Shows the safety outcome of hypotension. (**E**) Shows the safety outcome of hyperkalemia. (**F**) Shows a composite of primary or safety outcomes.

We used Cox proportional hazards models to generate the survival curves presented in Figure 2. These Cox adjusted survival curves account for multiple covariates, i.e., age, gender, body mass index, dialysis duration, comorbidities and baseline medications, providing a more accurate representation of the adjusted risk over time. Association between treatments and the outcomes of interest were examined by the model and presented as the hazard ratios (HR) and 95% confidence interval (CI). A detailed list of the adjusted factors is provided in Table 1. Changes in heart function measured by echocardiogram during follow-up in each group were compared using the paired *t*-test for continuous variables. The difference-in-difference analysis was used to compare the changes of echocardiographic parameters between the groups.

**Table 1:** All outcomes in the comparison between the ARNI and ACEI/ARB group.

	ARNI (*n* = 42)		ACEI/ARB (*n* = 47)		ARNI (*n* = 42)	ACEI/ARB (*n* = 47)	Adjusted HR^d^ (95% CI)	*P* value
Primary outcome	Events	(%)	Events	(%)	Events per 100 patient-year	Events per 100 patient-year		
HHF + all-cause mortality	6	(14.3)	10	(21.3)	20.6	26.1	0.98 (0.28–3.43)	0.97
Secondary outcomes								
HHF	3	(7.1)	5	(10.6)	10.3	13.0	1.24 (0.22–6.90)	0.81
All-cause mortality	3	(7.1)	5	(10.6)	10.3	13.0	0.81 (0.13–5.22)	0.83
Safety outcomes								
Hypotension ^a^	13	(31.0)	26	(55.3)	44.5	67.7	0.70 (0.30–1.62)	0.40
Hyperkalemia ^b^	12	(28.6)	26	(55.3)	41.1	67.7	0.46 (0.19–1.09)	0.08
Win ratio ^c^	21	(50.0)	36	(76.6)	71.9	93.8	0.73 (0.38–1.44)	0.37

ACEI, angiotensin converting enzyme inhibitor; ARB, angiotensin receptor blocker; ARNI, angiotensin receptor-neprilysin inhibitors; HHF, hospitalization for heart failure; HR, hazard ratio; CI, confidence interval.

^a^ Hypotension, systolic blood pressure less than 90 mmHg.

^b^ Hyperkalemia, serum potassium level more than 5 mmol/L.

^c^ Win ratio, the efficacy and safety of the ARNI group versus the ACEI/ARB group.

^d^ Adjusted factors: adjusted for age, gender, body mass index, dialysis duration, index date, comorbidities (coronary artery disease, stroke, diabetes, hypertension), and baseline medications (beta-blocker, mineralocorticoid receptor antagonist, ivabradine, and nitrate).

In this study, we utilized a win-ratio analysis to compare the effectiveness of ARNI vs. ACEI/ARB therapy in our patient cohort. The win-ratio analysis is a non-parametric approach that aggregates outcomes across multiple dimensions into a single measure. For our analysis, the outcomes were classified as follows: mortality, HHF, and safety outcomes, including hypotension and hyperkalemia. Each patient in the ARNI group was compared to each patient in the ACEI/ARB group, with wins tallied for each outcome. A patient is considered to have a “win” if their outcome is better than that of a counterpart from the comparison group.

We conducted sensitivity analyses to examine the outcome robustness of the clinical effects. First, the bootstrap methods for repetitive sampling 1,000 times were applied to consider the impact of sample size (18). Besides, a propensity score matching procedure was used to account for the heterogeneity and control for baseline confounding (19). Patients treated with ARNI were matched in a 1:1 ratio with those receiving ACEI/ARB. Variables used in the propensity score model included age, index date, baseline medications, and comorbidities. Given the small sample size, we adopted a less stringent matching approach using calibers of width equal to 0.6 to preserve most of the patient data. This decision resulted in some residual imbalances. To address this, we conducted further analysis using the residual imbalances as factors in a multivariable adjustment. Considering that a larger sample size might enhance the reliability and generalizability of the results, we conducted another sensitivity analysis, including the patients who had encountered outcome events within six months before the index date. Last, to account for the potentially inadequate period to capture cardiovascular outcomes, instead of the original 1-year censoring point, an extended follow-up period (until December 31, 2021) was applied for all patients. Independent Cox regression models were performed for each sensitivity analysis using the same methods as our primary analysis.

A *P*-value of <0.05 was considered statistically significant in this study. All statistical operations were executed using the Statistical Package for Social Sciences for Windows 17.0 (SPSS Inc., Chicago, IL) and SAS EG software (version 8.3; SAS Institute, Cary, NC).

## Results

### Baseline characteristics

The final analysis included 89 patients: 42 in the ARNI group (28 men, mean age 59.9 ± 12.4 years, LVEF 29.7 ± 7.5%, dialysis duration 4.8 ± 3.9 years) and 47 in the ACEI/ARB group (28 men, mean age 65.6 ± 10.1 years, LVEF 31.2 ± 6.1%, dialysis duration 4.4 ± 3.4 years) (Table 2). The ARNI group had a higher prevalence of coronary artery disease and stroke (45.2% vs. 27.7% and 14.3% vs. 4.3%, respectively). The median duration of ACEI or ARB use before the index date was comparable between both groups. Ivabradine and nitrates were used more frequently in the ARNI group compared to the ACEI/ARB group. There were no significant differences in baseline laboratory data between the two groups.

**Table 2:** Baseline characteristics of the included subjects.

	ARNI (*n* = 42)	ACEI/ARB (*n* = 47)	*P* value
Demographics			
Men, *n* (%)	28 (66.7)	28 (59.6)	0.49
Age, year	59.9 ± 12.4	65.6 ± 10.1	0.02*
Clinical characteristics			
Body mass index, kg/m2	23.9 ± 4.3	22.4 ± 3.8	0.11
Office mean systolic pressure, mmHg	140.0 ± 16.8	138.2 ± 19.4	0.65
Heart rate, beat per minute	85.6 ± 12.7	84.2 ± 16.4	0.64
Dialysis duration, year	4.8 ± 3.9	4.4 ± 3.4	0.64
LVEF, %	29.0 ± 7.7	31.5 ± 5.7	0.32
Medical history			
Hypertension, *n* (%)	33 (78.6)	41 (87.2)	0.28
Diabetes, *n* (%)	26 (61.9)	25 (53.2)	0.41
Coronary artery disease, *n* (%)	19 (45.2)	13 (27.7)	0.08
Atrial fibrillation, *n* (%)	1 (2.4)	1 (2.1)	0.94
Hospitalization for heart failure, *n* (%)	2 (4.8)	5 (10.6)	0.30
Myocardial infarction, *n (*%)	6 (14.3)	4 (8.5)	0.39
Stroke, *n* (%)	6 (14.3)	2 (4.3)	0.10
Pulmonary diseases, *n* (%)	9 (21.4)	11 (23.4)	0.82
Liver diseases, *n* (%)	10 (23.8)	7 (14.9)	0.29
Cancer, *n* (%)	4 (9.5)	6 (12.8)	0.63
Medications for comorbidities			
Receiving ACEI/ARB duration within 1 year before index date, day	33.5 (0–114.5)	42 (0–140)	0.62
Diuretics, *n* (%)	31 (73.8)	35 (74.5)	0.94
Digitalis, *n* (%)	6 (14.3)	12 (25.5)	0.19
Beta-blocker, *n* (%)	38 (90.5)	42 (89.4)	0.86
Mineralocorticoid receptor antagonist, *n* (%)	19 (45.2)	12 (25.5)	0.05
Ivabradine, *n* (%)	21 (50.0)	10 (21.3)	0.01*
SGLT2-inhibitors, *n* (%)	1 (2.4)	0 (0.0%)	0.29
Nitrate, *n* (%)	34 (81.0)	27 (57.4)	0.02*
Calcium polystyrene sulfonate *n* (%)	2 (4.8)	1 (2.1)	0.49
Mean laboratory data			
Albumin, g/dl	3.3 ± 0.5	3.5 ± 0.5	0.13
Potassium, mmol/L	4.0 ± 0.8	4.0 ± 0.6	0.64
Calcium, mg/dl	8.9 ± 1.0	8.9 ± 1.3	0.72
Phosphates, mg/dl	6.0 ± 2.8	5.3 ± 1.5	0.19
PTH-intact, pg/ml	283.4 (93.6–551.4)	390.9 (144.2–802.6)	0.82
Hemoglobin, g/dl	9.5 ± 1.3	9.9 ± 1.7	0.34
NT-proBNP, pg/ml	22,625 ± 6,047.9	23,741 ± 4,562.1	0.47
Iron, ug/dl	61.0 ± 24.3	59.2 ± 25.8	0.79
Ferritin, ng/ml	349.8 (251.6–552.4)	420.4 (256.0–547.1)	0.53

* *P* < 0.05 means reaching statistical significance.

Values are arithmetic mean ± SD or median (interquartile range).

ARNI, angiotensin receptor-neprilysin nhibitors; ACEI, angiotensin converting enzyme; ARB, angiotensin receptor blocker; LVEF, left ventricular ejection fraction; SGLT2, sodium–glucose cotransporter 2; PTH-intact, parathyroid hormone-intact.

### Clinical outcomes

After a 1-year follow-up, six events in primary outcomes including a composite of heart failure hospitalization or mortality (20.6 events per 100 patient-year) occurred in the ARNI group and ten (26.1 events per 100 patient-year) occurred in the ACEI/ARB group (adjusted HR 0.98, 95% CI: 0.28–3.43, *P* = 0.97) (Figure 2A and Table 1). The individual incidence of HHF was 10.3 and 13 events per 100 patient-years in the ARNI and the ACEI/ARB groups (adjusted HR 1.24, 95% CI: 0.22–6.90, *P* = 0.81), and the all-cause mortality were 10.3 and 13 (adjusted HR 0.81, 95% CI: 0.13–5.22, *P* = 0.83) (Figures 2B,C). Regarding safety outcomes, there were 13 and 26 hypotension events in ARNI and ACEI/ARB groups, and the incidence were 44.5 vs. 67.7 per 100 patient-year (adjusted HR 0.70, 95% CI: 0.30–1.62, *P* = 0.40). Furthermore, the ARNI group had 12 hyperkalemia events and the ACEI/ARB group had 26 events. The incidences were 41.1 vs. 67.7 (adjusted HR 0.46, 95% CI: 0.19–1.09, *P* = 0.08) (Table 1; Figures 2D,E). The win-ratio analysis provided a holistic view of the treatment effects by comparing multiple outcomes simultaneously; the ARNI group compared to the ACEI/ARB group was calculated to be 0.73 (95% CI: 0.38–1.44, *P* = 0.83), indicating no statistically significant advantage for the ARNI group in this cohort. Specifically, the analysis included mortality, HHF, and safety outcomes (hypotension and hyperkalemia). After integrating primary and safety outcomes, the adjusted HR of the composite efficacy or safety outcomes was 0.73 (95% CI: 0.38–1.44, *P* = 0.37) (Figure 2F).

### Echocardiogram parameters

After 1 year of treatment, the significant changes of echocardiographic parameters in the ARNI group were LVEF (29.0 ± 7.7% vs. 37.6 ± 9.5%, *P* < 0.01) and LVIDs (5.2 ± 0.6 vs. 4.9 ± 0.7 cm, *P* < 0.001), while the ACEI/ARB groups had significant changes in LVEF (31.5 ± 5.7% vs. 42.2 ± 12.6%, *P* < 0.01), LVIDd (5.9 ± 0.8 vs. 5.6 ± 0.9 cm, *P* = 0.045) and LVIDs (4.9 ± 0.7 vs. 4.4 ± 1.1 cm, *P* = 0.004) (Table 3). The difference-in-difference analysis showed that the changes of these parameters after 1-year treatment between the groups were not significantly different.

**Table 3:** Changes in echocardiography parameters.

Echocardiography parameters	ARNI (*n* = 42)				ACEI/ARB (*n* = 47)				
	Baseline	Follow-up	Difference	*P* value	Baseline	Follow-up	Difference	*P* value	*P* value*
LVEF, %	29.0 ± 7.7	37.6 ± 9.5	8.56 ± 9.8	<0.010	31.5 ± 5.7	42.2 ± 12.6	10.71 ± 12.7	<0.010	0.39
LVIDd, cm	6.0 ± 0.7	5.9 ± 0.7	−0.10 ± 0.5	0.233	5.9 ± 0.8	5.6 ± 0.9	−0.30 ± 1.0	0.045	0.24
LVIDs, cm	5.2 ± 0.6	4.9 ± 0.7	−0.30 ± 0.6	<0.001	4.9 ± 0.7	4.4 ± 1.1	−0.52 ± 1.2	0.004	0.31
LAD, cm	4.5 ± 0.6	4.4 ± 0.6	−0.04 ± 0.8	0.748	4.5 ± 0.7	4.3 ± 0.8	−0.11 ± 0.8	0.305	0.66

ACEI, angiotensin converting enzyme inhibitor; ARB, angiotensin receptor blocker; ARNI, angiotensin receptor-neprilysin inhibitors; LVEF, left ventricular ejection fraction; LVIDd, left ventricular internal diameter end diastole; LVIDs, left ventricular internal diameter end systole; LAD, left atrial Diameter.

* The difference in the change between the ARNI and ACEI/ARB group.

### Sensitivity analyses

The analysis with the bootstrap method showed consistency in the primary, secondary, and safety outcomes (Supplementary Table S1). If we carried out the propensity score matching procedure before the analysis, the HR of total HHF or mortality in ARNI vs. ACEI/ARB became 0.91 (95% CI: 0.31–2.62, *P* = 0.86) (Supplementary Figure S1). As we included 37 patients, who were initially excluded due to early outcomes within 6 months before the index date, the adjusted HR of the primary outcomes was 0.66 (95% CI: 0.28–1.55, *P* = 0.34) (Supplementary Figure S2). In the more extended follow-up analysis, the median follow-up period was 1.52 years and 2.68 years for ARNI and ACEI/ARB. The incidence of primary outcomes was 12.6 and 9.5 events per 100 patient-year in the ARNI and the ACEI/ARB groups (adjusted HR 1.28, 95% CI: 0.43–3.84, *P* = 0.66) (Supplementary Table S2).

## Discussion

The well-established benefits of ARNI in HFrEF patients are promising, yet the treatment effects in those with advanced CKD remain unclear. In our multi-institutional study involving individuals with ESRD undergoing dialysis, we discovered that the ARNI group likely exhibited statistically similar cardiovascular benefits compared to the ACEI/ARB group, with outcomes remaining consistent across various sensitivity analyses. Importantly, while the advantageous effects of ARNI may be less pronounced in patients with both HFrEF and ESRD undergoing dialysis, the ARNI group showed a trend towards lower risks of hypotension and hyperkalemia. This suggests that ARNI could be a viable alternative with safety considerations. Our findings may offer valuable insights for clinicians seeking to optimize treatments while taking into account financial constraints, adverse effects, and patient preferences.

Numerous real-world studies have explored the effectiveness of ARNI in patients with concomitant HFrEF and advanced CKD (10–12). Chang et al. demonstrated that the benefits of ARNI in reducing the risk of cardiovascular death and HHF were consistent across various CKD stages before ESRD and dialysis initiation among HFrEF patients (10). However, other studies involving patients with advanced CKD and ESRD undergoing dialysis revealed negligible benefits (11, 12). Intriguingly, Chang et al. showed that the treatment effect in mortality reduction was comparable between both groups in their subgroup analysis. Another subgroup analysis within these studies, which included a majority of patients with ESRD undergoing dialysis (59.5%), indicated an association between the ARNI group and a higher risk of HHF (11). These findings implied that the treatment advantages of ARNI could wane in this specific population. Our study, specifically targeting HFrEF individuals with ESRD and dialysis, identified insignificant difference in outcomes between the ARNI and ACEI/ARB groups, thus corroborating the observations made in previous research.

There are plausible reasons for these findings. The treatment of HFrEF patients with ARNI has been reported to benefit the preservation of residual renal function (5, 20). This advantage is likely crucial for cardiovascular protection due to its positive effects on solute and uremic toxins clearance, and amelioration of anemia, chronic inflammation, valvular calcification, atherosclerosis, and cardiac hypertrophy (21, 22). However, these biological advantages are typically minimal in those with ESRD undergoing dialysis. This reduction in benefits can be attributed to the limited residual renal function in these patients, which may not be sufficient to generate meaningful clinical improvements (22, 23). Furthermore, dialysis itself may induce hemodynamic changes and oxidative stress, which could counteract the beneficial effects of ARNI on cardiovascular outcomes (24, 25). Collectively, this may explain the insignificant association between ARNI and lower risk of HHF and mortality for those receiving ARNI with ESRD in these observational studies.

Despite the insignificant association for individuals receiving ARNI, a closer examination of the data reveals notable improvements in LVEF and LVIDs within the ARNI group. These improvements are significant and align with the known benefits of ARNI in enhancing cardiac function (13). In previous real-world studies, ARNI use contributed to improved LVEF in HFrEF patients after 1-year follow-up, regardless of their dialysis status (11–13). For example, a case-control study involving 49 HFrEF patients demonstrated the benefits in both hemodialysis and peritoneal dialysis groups (13). This phenomenon may result from LVEF improvement and afterload reduction in the ESRD population. Notably, even though blood pressure is commonly used as a surrogate for afterload, we did not observe a higher incidence of hypotension events in the ARNI group compared to the ACEI/ARB group. These observed findings suggests that ARNI may offer substantial benefits in cardiac remodeling and function in patients with HFrEF undergoing dialysis. However, it is important to balance these findings with the overall outcomes and consider the need for larger and longer-term studies to further validate these benefits.

For safety, the ARNI group demonstrated a trend towards fewer hyperkalemia events. The trend may be partially explained by the pharmacodynamic properties of ARNI, mainly through its component Neprilysin. Neprilysin potentially increases renal blood flow by facilitating the dilation of the glomerular afferent arteriole. This increase could enhance renal potassium excretion, particularly in dialysis patients who retain some level of renal function. Additionally, both Hsiao et al. and our study found comparable LVEF improvements between the ARNI and ACEI/ARB groups (11), while Chang et al. reported a significantly better treatment effect in the ARNI group (12). Future studies are warranted to further examine the treatment effects on reverse cardiac remodeling between both groups in HFrEF patients with ESRD undergoing dialysis.

There are some limitations in this study. First, the sample size and a lower risk of patient profile might influence the examination of statistical differences between the groups. To address this issue, we conducted further analysis using the bootstrap method and including the patients with early outcome events prior the index date to account for this weakness. Although the outcomes remained unchanged, the effectiveness of ARNI in this population still requires cautious interpretation. Second, the follow-up period in this study was 1 year, which might be insufficient to capture all cardiovascular outcomes. Nevertheless, the sensitivity analysis with an extended follow-up still showed non-significantly different outcomes between both groups. Another limitation was the exclusion of NT-pro-BNP levels as an outcome measure. NT-pro-BNP levels are significantly influenced by hemodialysis conditions in ESRD patients, which can lead to fluctuations that do not accurately reflect cardiac function in this population. Additionally, routine follow-up of NT-pro-BNP in stable HFrEF patients is not recommended according to current guidelines. Reimbursement for NT-pro-BNP testing may also not be permitted under our national healthcare insurance policy. These factors make NT-pro-BNP an unsuitable outcome measurement for this study. Last, inherent limitations of our retrospective study design may introduce biases and affect the reliability of our data collection and analysis compared to prospective studies. To address these concerns, we employed several strategies to mitigate potential biases. Robust statistical methods, including propensity score matching, were used to balance baseline characteristics between the ARNI and ACEI/ARB groups. We also performed sensitivity analyses to ensure the robustness of our results. Multiple imputations were conducted for missing data, and various covariates were adjusted for in our Cox proportional hazards models. Nevertheless, although we have done our best to control for bias and confounding, it is impossible to control all confounding, and residual unmeasurable confounding might interfere with the outcomes. Therefore, we recommend that future randomized controlled trials be conducted to provide stronger evidence on the treatment effects of ARNI in patients with HFrEF undergoing dialysis.

## Conclusion

This study found that among HFrEF patients with ESRD undergoing dialysis, the ARNI group probably had statistically similar cardiovascular benefits and safety to the ACEI/ARB group. Despite the lack of statistical significance, the trend towards lower risks of hypotension and hyperkalemia in the ARNI group suggested a potentially favorable safety profile in this high-risk patient group. The findings contribute to the growing body of evidence on the effectiveness of ARNI in real-world settings among these patients. Further large-scale prospective studies are warranted to confirm our findings and explore the potential benefits of ARNI in this specific population.

## Data Availability

The raw data supporting the conclusions of this article will be made available by the authors, without undue reservation.
