# Supplementary material for: Real-world experience of angiotensin receptor-neprilysin inhibitors in patients with heart failure and dialysis
Source: Front Cardiovasc Med. 2024 Jul 22;11:1393440. doi: 10.3389/fcvm.2024.1393440 (PMC11298400; doi:10.3389/fcvm.2024.1393440)
Supplement: Supplementary file 1 [file Datasheet1.docx]

**Supplementary Material**

**Real-world Experience of Angiotensin Receptor-Neprilysin Inhibitors in Patients with Heart Failure and Dialysis**

I-Ning Yang^*^, Chi-Ya Huang, Chun-Ting Yang, Han-Siong Toh, Wei-Ting Chang, Li-Wei Su, Yu-Min Lin, Ming-Cheng Wang, Hsien-Yi Wang, Chia-Te Liao^*^

^*^**Correspondence:** Corresponding authors:

Chia-Te Liao ^*^

Email: [drctliao@gmail.com](mailto:drctliao@gmail.com)

I-Ning Yang^*^

Email: [inyang5036@gmail.com](mailto:inyang5036@gmail.com)

**Supplementary Material Content**

**Supplementary Methods**

**Supplementary Tables**

**Supplementary Table S1.** All outcomes with bootstrap in the comparison between the ARNI and ACEI/ARB group.

**Supplementary Table S2.** Primary and secondary outcomes (Duration until December 31, 2021) in the comparison between the ARNI and the ACEI/ARB group.

**Supplementary Table S3.** Baseline characteristics of the included subjects with propensity score matching.

**Supplementary Table S4.** Number at Risk Over Time with Cox-Adjusted Cumulative Event-Free Rates for Primary Composite Outcomes

**Supplementary Table S5.** Number at Risk Over Time with Cox-Adjusted Cumulative Event-Free Rates for Hospitalization Due to Heart Failure

**Supplementary Table S6.** Number at Risk Over Time with Cox-Adjusted Cumulative Event-Free Rates for All-cause Mortality

**Supplementary Figures**

**Supplementary Figure S1.** Cox-adjusted cumulative event-free rate of primary composite outcome with propensity score matching.

**Supplementary Figure S2.** Cox-adjusted cumulative event-free rate of primary composite outcome with patients initially excluded for prior outcome events

**Supplementary Files**

**Supplementary File S1.** ICD-9-CM and ICD-10-CM codes used to identify clinical conditions and outcomes.

| **Supplementary Table S1. All outcomes with bootstrap in the comparison between the ARNI and the ACEI/ARB group** | | | | | | | | |
| --- | --- | --- | --- | --- | --- | --- | --- | --- |
|  | **ARNI (n = 42)** | | **ACEI/ARB (n = 47)** | | **ARNI (n = 42)** | **ACEI/ARB (n = 47)** | **Adjusted HR (95% CI)** | **P value** |
| Primary outcome | Events | (%) | Events | (%) | Events per 100 patient-year | Events per 100 patient-year |  |  |
| HHF + all-cause death | 6 | (14.3) | 10 | (21.3) | 20.6 | 26.1 | 0.98 (0.28-3.43) | 0.97 |
| Secondary outcomes |  | | | | | | | |
| HHF | 3 | (7.1) | 5 | (10.6) | 10.3 | 13.0 | 1.24 (0.22-6.91) | 0.81 |
| All-cause death | 3 | (7.1) | 5 | (10.6) | 10.3 | 13.0 | 0.81 (0.13-5.22) | 0.83 |
| Safety outcomes |  | | | | | | | |
| Hypotension | 13 | (31.0) | 26 | (55.3) | 44.5 | 67.7 | 0.70 (0.30-1.62) | 0.40 |
| Hyperkalemia | 12 | (28.6) | 26 | (55.3) | 41.1 | 67.7 | 0.46 (0.19-1.09) | 0.08 |
| Hypotension, defined as systolic blood pressure less than 90mmHg  Hyperkalemia, defined as serum potassium level more than 5mmol/L  ACEI, angiotensin converting enzyme inhibitor; ARB, angiotensin receptor blocker; ARNI, angiotensin receptor-neprilysin inhibitor;  HHF, hospitalization for heart failure; HR, hazard ratio; CI, confidence interval | | | | | | | | |

| **Supplementary Table S2. Primary and secondary outcomes (Duration until December 31, 2021) in the comparison between the ARNI and the ACEI/ARB group** | | | | | | | | |
| --- | --- | --- | --- | --- | --- | --- | --- | --- |
|  | **ARNI (n = 42)** | | **ACEI/ARB (n = 47)** | | **ARNI (n = 42)** | **ACEI/ARB (n = 47)** | **Adjusted HR (95% CI)** | **P value** |
| Primary outcome | Events | (%) | Events | (%) | Events per 100 patient-year | Events per 100 patient-year |  |  |
| HHF + all-cause death | 8 | (19.0) | 12 | (25.5) | 12.6 | 9.5 | 1.28 (0.43-3.84) | 0.66 |
| Secondary outcomes |  | | | | | | | |
| HHF | 5 | (11.9) | 6 | (12.8) | 7.9 | 4.8 | 2.32 (0.52-10.42) | 0.27 |
| All-cause death | 3 | (7.1) | 5 | (10.6) | 4.7 | 4.0 | 0.70 (0.11-4.45) | 0.70 |
| Hypotension, defined as systolic blood pressure less than 90mmHg  Hyperkalemia, defined as serum potassium level more than 5mmol/L  ACEI, angiotensin converting enzyme inhibitor; ARB, angiotensin receptor blocker; ARNI, angiotensin receptor-neprilysin inhibitor;  HHF, hospitalization for heart failure; HR, hazard ratio; CI, confidence interval  Adjusted factors: adjusted for age, gender, body mass index, dialysis duration, index date, comorbidities (coronary artery disease, stroke, diabetes, hypertension), and baseline medications (beta-blocker, mineralocorticoid receptor antagonist, ivabradine, and nitrate). | | | | | | | | |

| **Supplementary Table S3. Baseline characteristics of the included subjects with propensity score matching** | | | | | | | | |  |  |
| --- | --- | --- | --- | --- | --- | --- | --- | --- | --- | --- |
|  | **ARNI (n = 41)** | | | **ACEI/ARB (n = 41)** | | | **P value** | | |  |
| Demographics |  | | |  | | |  | | |  |
| Men, n(%) | 27 | (65.9) | | 24 | (58.5) | | 0.49 |  | |  |
| Age, y | 59.8 | ± | 12.5 | 65.1 | ± | 10.1 | 0.04 | ^a^ | |  |
| Clinical characteristics |  |  |  |  |  |  |  |  | |  |
| Body mass index, kg/m^2^ | 22.6 | ± | 4.4 | 23.9 | ± | 3.8 | 0.18 |  | |  |
| Office mean systolic pressure, mmHg | 139.6 | ± | 16.8 | 136.8 | ± | 18.7 | 0.47 |  | |  |
| Heart rate, beat per minute | 85.3 | ± | 12.7 | 84.3 | ± | 16.7 | 0.75 |  | |  |
| Dialysis duration, y | 4.9 | ± | 3.9 | 4.5 | ± | 3.4 | 0.58 |  | |  |
| LVEF, % | 30.1 | ± | 7.2 | 30.8 | ± | 6.0 | 0.61 |  | |  |
| Medical history |  |  |  |  |  |  |  |  | |  |
| Hypertension, n(%) | 33 | (78.6) | | 41 | (87.2) | | 0.28 |  | |  |
| Diabetes, n(%) | 26 | (63.4) | | 25 | (60.9) | | 0.82 |  | |  |
| Coronary artery disease, n(%) | 19 | (46.3) | | 13 | (31.7) | | 0.17 |  | |  |
| Atrial fibrillation, n(%) | 1 | (2.4) | | 1 | (2.4) | | 1.00 |  | |  |
| Hospitalization for heart failure, n(%) | 2 | (4.9) | | 5 | (12.2) | | 0.24 |  | |  |
| Myocardial infarction, n(%) | 6 | (14.6) | | 4 | (9.7) | | 0.50 |  | |  |
| Stroke, n(%) | 6 | (14.6) | | 2 | (4.9) | | 0.14 |  | |  |
| Pulmonary diseases, n(%) | 9 | (22.0) | | 11 | (26.8) | | 0.61 |  | |  |
| Liver diseases, n(%) | 10 | (24.4) | | 7 | (17.1) | | 0.41 |  | |  |
| Cancer, n(%) | 4 | (9.7) | | 6 | (14.6) | | 0.50 |  | |  |
| Medications for comorbidities |  | | | | | | | | |  |
| Receiving ACEI/ARB duration within one   year before index date, d | 72.2 | (0-115.0) | | 83.7 | (0-143) | | 0.59 |  | |  |
| Diuretics, n(%) | 30 | (73.2) | | 33 | (80.5) | | 0.43 |  | |  |
| Digitalis, n(%) | 6 | (14.6) | | 11 | (26.8) | | 0.17 |  | |  |
| Beta-blocker, n(%) | 37 | (90.2) | | 38 | (92.7) | | 0.69 |  | |  |
| Mineralocorticoid receptor antagonist,  n(%) | 18 | (43.9) | | 12 | (29.3) | | 0.17 |  | |  |
| Ivabradine, n(%) | 20 | (48.8) | | 10 | (24.4) | | 0.02 | ^a^ | |  |
| SGLT2-inhibitors, n(%) | 1 | (2.4) | | 0 | (0.0%) | | 0.31 |  | |  |
| Nitrate, n(%) | 33 | (80.0) | | 25 | (61.0) | | 0.05 |  | |  |
| Calcium polystyrene sulfonate n(%) | 1 | (2.4) | | 1 | (2.4) | | 1.00 |  | |  |
| Mean laboratory data |  |  |  |  |  |  |  |  | |  |
| Albumin, g/dl | 3.3 | ± | 0.5 | 3.4 | ± | 0.5 | 0.09 |  | |  |
| Potassium, mmol/l | 4.0 | ± | 0.7 | 4.0 | ± | 0.6 | 0.76 |  | |  |
| Calcium, mg/dl | 8.9 | ± | 1.0 | 8.9 | ± | 1.1 | 0.83 |  | |  |
| Phosphates, mg/dl | 6.0 | ± | 2.8 | 5.2 | ± | 1.5 | 0.17 |  | |  |
| PTH-Intact, pg/ml | 442.0 | (86.3-597.0) | | 447.1 | (114.2-828.5) | | 0.79 |  | |  |
| Hemoglobin, g/dl | 9.5 | ± | 1.3 | 9.7 | ± | 1.7 | 0.60 |  | |  |
| NT-proBNP, pg/ml | 22625 | ± | 6047.9 | 23454 | ± | 5030.4 | 0.63 |  | |  |
| Iron, ug/dl | 61.1 | ± | 24.7 | 57.6 | ± | 24.5 | 0.59 |  | |  |
| Ferritin, ng/ml | 571.1 | (251.2-574.2) | | 482.5 | (252.2-548.1) | | 0.54 |  | |  |
| ^a^ *P* < 0.05 means reaching statistical significance  Values are arithmetic mean ± SD or median (interquartile range)  ARNI, angiotensin receptor-neprilysin inhibitor; ACEI, angiotensin converting enzyme inhibitor;  ARB, angiotensin receptor blocker; LVEF, left ventricular ejection fraction;  SGLT2, sodium-glucose cotransporter 2; PTH-Intact, parathyroid hormone-intact | | | | | | | | | | |

| **Supplementary Table S4. Number at Risk Over Time with Cox-Adjusted Cumulative Event-Free Rates for Primary Composite Outcomes** | | | | | | | | | | | | | |
| --- | --- | --- | --- | --- | --- | --- | --- | --- | --- | --- | --- | --- | --- |
| Day | 0 | 30 | 60 | 90 | 120 | 150 | 180 | 210 | 240 | 270 | 300 | 330 | 360 |
| AA, n (N) | 0 | 1 (47) | 4 (43) | 1 (42) | 0 (42) | 0 (41) | 0 (39) | 0 (38) | 0 (38) | 1 (37) | 2 (34) | 1 (32) | 0 (31) |
| ARNI, n (N) | 0 | 3 (38) | 0 (36) | 0 (36) | 1 (34) | 0 (32) | 0 (31) | 1 (28) | 1 (25) | 0 (23) | 0 (23) | 0 (23) | 0 (22) |
| Total, n (N) | 0 | 4 (85) | 4 (78) | 1 (77) | 1 (75) | 0 (72) | 0 (70) | 1 (65) | 1 (62) | 1 (59) | 2 (56) | 1 (54) | 0 (52) |
| n: Number at risk at the start of each time point (i.e., those still being followed and who have not yet experienced the event) N: Number of events occurring at the time point (referring here to primary composite outcomes) | | | | | | | | | | | | | |

| **Supplementary Table S5. Number at Risk Over Time with Cox-Adjusted Cumulative Event-Free Rates for Hospitalization Due to Heart Failure** | | | | | | | | | | | | | |
| --- | --- | --- | --- | --- | --- | --- | --- | --- | --- | --- | --- | --- | --- |
| Day | 0 | 30 | 60 | 90 | 120 | 150 | 180 | 210 | 240 | 270 | 300 | 330 | 360 |
| AA, n (N) | 0 | 0 (47) | 3 (43) | 1 (42) | 0 (42) | 0 (41) | 0 (39) | 0 (38) | 0 (38) | 1 (37) | 0 (34) | 0 (32) | 0 (31) |
| ARNI, n (N) | 0 | 2 (38) | 0 (36) | 0 (36) | 0 (34) | 0 (32) | 0 (31) | 1 (28) | 0 (25) | 0 (23) | 0 (23) | 0 (23) | 0 (22) |
| Total, n (N) | 0 | 2 (85) | 3 (78) | 1 (77) | 0 (75) | 0 (72) | 0 (70) | 1 (65) | 0 (62) | 1 (59) | 0 (56) | 0 (54) | 0 (52) |
| n: Number at risk at the start of each time point (i.e., those still being followed and who have not yet experienced the event) N: Number of events occurring at the time point (referring here to hospitalization for heart failure) | | | | | | | | | | | | | |

| **Supplementary Table S6. Number at Risk Over Time with Cox-Adjusted Cumulative Event-Free Rates for All-cause Mortality** | | | | | | | | | | | | | |
| --- | --- | --- | --- | --- | --- | --- | --- | --- | --- | --- | --- | --- | --- |
| Day | 0 | 30 | 60 | 90 | 120 | 150 | 180 | 210 | 240 | 270 | 300 | 330 | 360 |
| AA, n (N) | 0 | 1 (47) | 1 (46) | 0 (46) | 0 (46) | 0 (46) | 0 (46) | 0 (46) | 0 (46) | 0 (46) | 2 (44) | 1 (43) | 0 (43) |
| ARNI, n (N) | 0 | 0 (42) | 0 (42) | 0 (42) | 0 (42) | 0 (42) | 0 (42) | 0 (42) | 1 (42) | 0 (42) | 0 (42) | 0 (42) | 0 (42) |
| Total, n (N) | 0 | 1 (89) | 1 (88) | 0 (88) | 0 (88) | 0 (88) | 0 (88) | 0 (88) | 1 (87) | 0 (87) | 2 (85) | 1 (84) | 0 (84) |
| n: Number at risk at the start of each time point (i.e., those still being followed and who have not yet experienced the event) N: Number of events occurring at the time point (referring here to all-cause mortality) | | | | | | | | | | | | | |

**Supplementary Figure S1. Cox-adjusted cumulative event-free rate of primary composite outcome with propensity score matching**

**
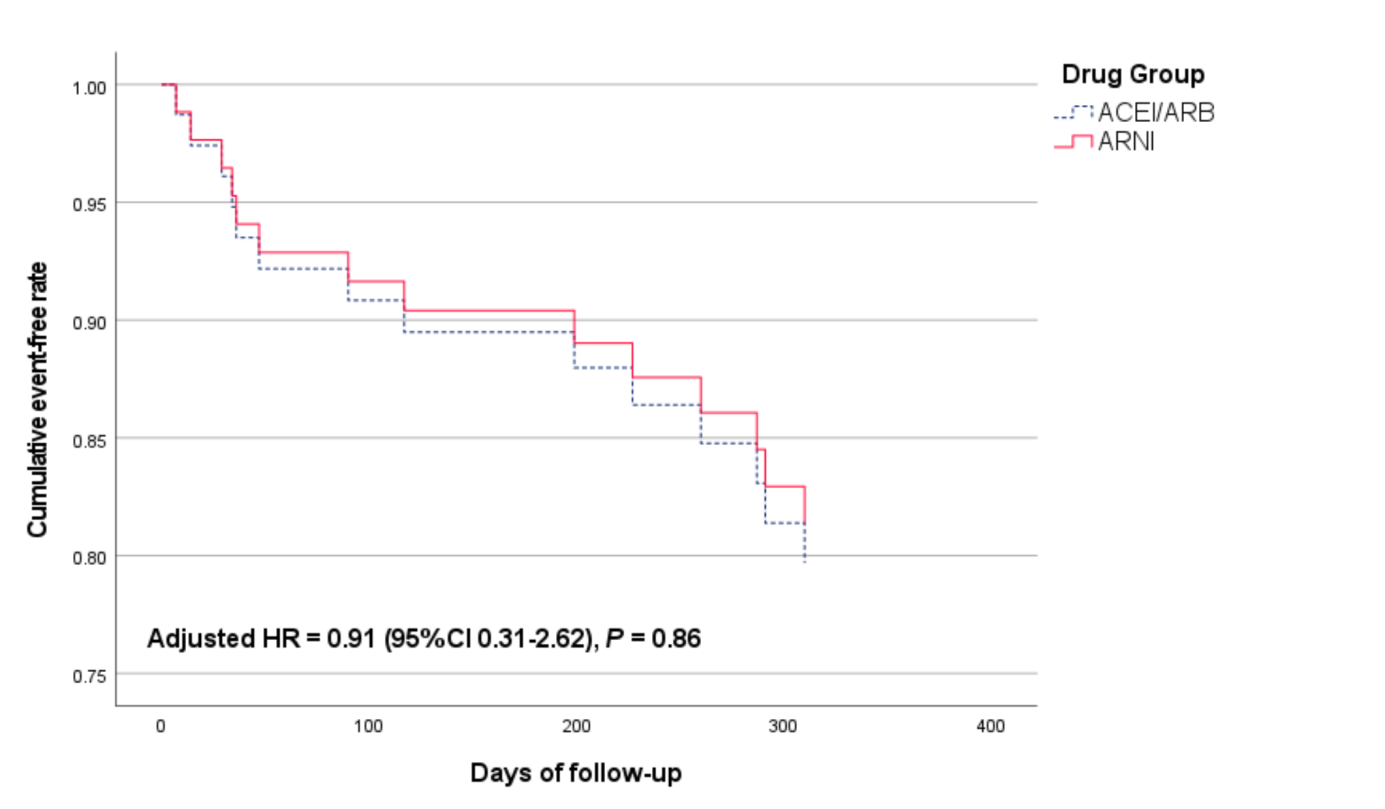
**

**Supplementary Figure S2. Cox-adjusted cumulative event-free rate of primary composite outcome with patients initially excluded for prior outcome events**


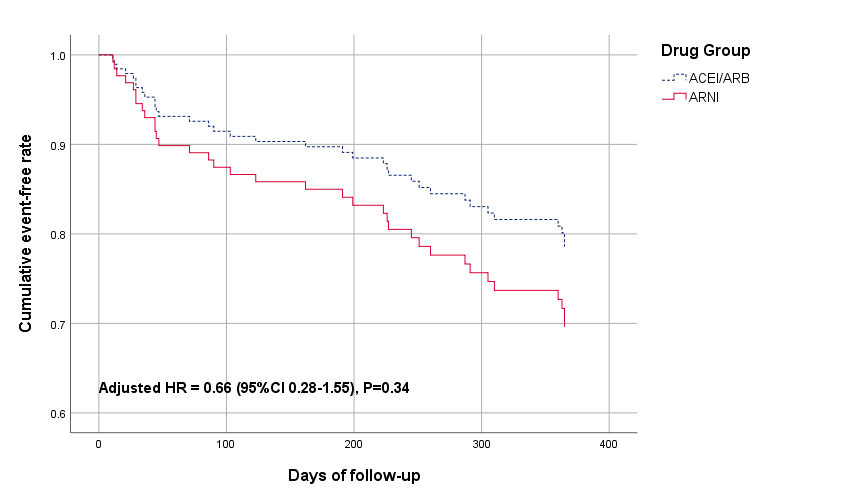


| **Supplementary File S1. ICD-9-CM and ICD-10-CM diagnosis codes used to identify clinical conditions and outcomes** | | |
| --- | --- | --- |
| **Conditions** | **ICD-9-CM** | **ICD-10-CM** |
| Hypertension | 401-405 | I10, I15 |
| Diabetes | 250, 357.2, 362.0, 366.41 | E08, E10, E11, E13 |
| Coronary artery disease | 410-414 | I25.1 |
| Atrial fibrillation | 427.3 | I48.0, I48.1, I48.2, I48.9, I48.91 |
| Hospitalization for heart failure | 398.91, 428, 402.11. 402.91 | I50.1, I50.2, I50.3, I50.4, I50.8, I50.9, I09.81, I11, I13 |
| Myocardial infarction | 410, 411, 412 | I21, I22, I23 |
| Stroke | 430-438 | G46.3, G46.4, I62.9, I63.01, I63.03, I63.2, I63.3, I63.4, I63.5, I63.8, I63.9, I69.3 |
| Pulmonary diseases | 491-494, 496, 510 | J44.1, J44.9, J45 |
| Liver diseases | 570, 571, 572.1, 572.4, 573.1-573.3 | B15-19, K70, K72-76 |
| Cancer | 140-172; 174-208; 230-231; 233-234 | C00-D49 |
| ICD-9-CM, International Classification of Diseases, 9^th^ Revision, Clinical Modification; ICD-10-CM, International Classification of Diseases, 10^th^ Revision, Clinical Modification | | |
